# Concurrent Hepatic Artery and Portal Vein Thrombosis after Orthotopic Liver Transplantation with Preserved Allografts

**DOI:** 10.1155/2014/384295

**Published:** 2014-04-10

**Authors:** Arshad Khan, P. Park, Jose Oberholzer, Ivo Tzvetanov, Raquel Garcia Roca, Ron C. Gaba, Enrico Benedetti, Hoonbae Jeon

**Affiliations:** ^1^Department of Surgery, College of Medicine, University of Illinois at Chicago, Chicago, IL 60612, USA; ^2^Department of Surgery, Korea University Guro Hospital, 80 Guro-dong, Guro-gu, Seoul 152-703, Republic of Korea; ^3^Department of Radiology, College of Medicine, University of Illinois at Chicago, Chicago, IL 60612, USA; ^4^Division of Transplantation, Department of Surgery, College of Medicine, University of Illinois at Chicago, CSB Suite 402, MC 958, 840 South Wood Street, Chicago, IL 60612, USA

## Abstract

In contrast to early HAT, late HAT has an insidious clinical presentation. Nevertheless, biliary and vascular reconstructions in this late setting are unlikely to improve outcome. Patent portal flow makes an important contribution to the viability of liver in case of late HAT while the allograft reconstitutes intrahepatic arterial flow through neovascularization. Concurrent HAT with PVT without immediate graft necrosis is extremely rare, and allograft and patient survival are seemingly impossible without retransplantation. In fact, hepatopetal arterial and portal venous neovascularization are known albeit obscure phenomena that can preserve posttransplant hepatic function under the extenuating circumstances of complete interruption of blood flow to the graft. We describe two such cases that developed combined HAT and PVT more than six months after OLT with perfect preservation of graft function. The survival of allografts in our cases was due to extensive hepatopetal arterial and portal venous collateralization. Simultaneous HAT and PVT after OLT are rare events and almost uniformly fatal, if they occur early. Due to paucity of such cases, however, underlying mechanisms and etiology remain elusive, and despite radiological diagnosis of these complications, there is no way to predict these events in the wake of stable graft function.

## 1. Introduction

The critical determinants of a successful liver transplant procedure are establishment of uncompromised inflow and adequate venous outflow. However, the postoperative course can be marred by vascular complications involving the HA and PV which are associated with high morbidity and mortality [1].

Although HAT and PVT occur infrequently, they pose a major threat to patient and graft survival and present formidable challenges for prevention and treatment. HAT has an incidence of 4–15% and when present in isolation may lead to acute graft failure and biliary destruction requiring retransplantation in most cases. Mortality may be as high as 27–58% and may increase to 73% when retransplantation is not carried out [2].

PVT complicates 2% of liver transplants and also can be fatal. It can present as portal hypertension, elevated transaminases, and acute graft failure [3]. While an increase in HA flow routinely accompanies a decrease in PV flow, the opposite does not characteristically occur and presents important implications in case of vascular compromise.

In the event of hepatic artery compromise the portal vein is the main source of hepatic blood flow and the liver responds by extracting greater amount of oxygen from portal blood and inducing neovascularization while viability is maintained by portal blood flow. Isolated portal vein thrombosis has been shown to cause hepatic infarction in the absence of arterial compromise [4].

If not recognized in timely manner, PVT leads to graft failure and retransplantation or patients death [5].

However, the biliary ductal system is solely supplied by HA via peribiliary plexus (PBP). Recent reports have indicated important contribution by portal circulation, as ischemic type biliary lesions (ITBL) develop in patients with a patent hepatic artery and thrombosed PV, implying altered microcirculation in PBP in face of intact arterial supply. This suggests that portal blood flow, while not only supplying hepatocytes, may make an important contribution to the biliary microcirculation and that compromised portal venous blood supply alone can predispose to the development of ITBL which necessitates retransplantation in 50% of such cases [6]. Thus, the HA and PV interplay in a harmonious way to preserve the hepatic viability when arterial or venous flow is jeopardized.

Given this fact it can be easily conceived that concurrent early HAT and PVT will have a devastating effect on biliary ductal system and hepatocytes necessitating transplantation. This notion is supported by few case reports in the literature showing prohibitive mortality in combined HAT and PVT [7]. There is not much information about the outcome of late concurrent HAT and PVT on the allograft due to extreme paucity of such cases in the literature.

With this in mind, we herein present two unique cases of concurrent late HAT and PVT in which patients survived many years after transplantation with normal graft function. The survival of the allograft in the present cases was due to extensive hepatopetal arterial and portal venous neovascularization.

## 2. Case Reports

**Case 1**

A 47-year-old female with a diagnosis of unresectable hilar cholangiocarcinoma had undergone biliary stents placement, neoadjuvant chemoradiation, brachytherapy, and maintenance chemotherapy according to the Mayo protocol. She subsequently underwent OLT. Concomitant pancreatoduodenectomy with native hepatectomy was performed to completely excise the distal bile duct possibly bearing malignancy and to approach the nonirradiated segment of the portal vein. The anastomosis of the portal vein was performed at the level of confluence between superior mesenteric and splenic veins in end-to-end fashion. The hepatic artery was reconstructed through an infrarenal aortic conduit using a donor iliac artery and the common hepatic artery of the recipient was ligated to avoid anastomosis to irradiated inflow vessel. After that, the continuity of pancreatobiliary and gastrointestinal tracts was restored.

On the 7th postoperative day, acute PVT was diagnosed on duplex US and triple phase CT, with subsequent confirmation by mesenteric angiography. Portal vein thrombectomy and anastomotic revision were urgently performed. There was no evidence of ischemic damage to the allograft. A thorough evaluation for hypercoagulable condition did not reveal any abnormalities and empirical anticoagulation with warfarin was started.

**Figure 1:**
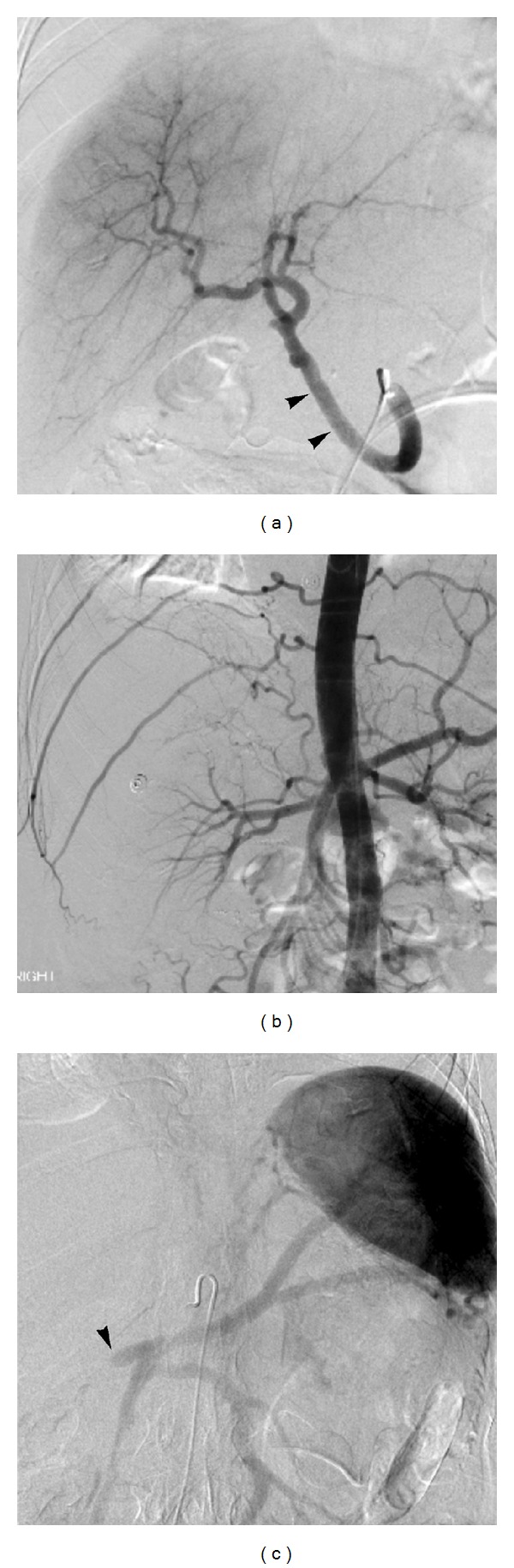
Digital subtraction angiogram (DSA) (a) shows patent iliac artery to hepatic artery graft (arrowheads). Digital subtraction aorta gram (b) demonstrates lack of iliac artery to hepatic artery graft opacification, indicating occlusion. Delayed venous phase imaging (c) reveals concurrent proximal portal vein occlusion (arrowhead).

Six months after transplantation, the patient presented to the hospital with vague diffuse abdominal pain despite all normal laboratory values and therapeutic INR of 2.5 on warfarin, and surveillance DUS discovered PVT and lack of extra hepatic arterial flow. On CT, despite totally obstructive HAT and PVT, the graft liver was well enhanced without perfusion defect or infarcted area. Angiography revealed multiple arterial collaterals supplying the graft. Collaterals from right intercostal arteries, a right adrenal artery, and jejunal branches from superior mesenteric artery were confirmed with angiogram as shown in Figures 1(a), 1(b), and 1(c). Three years after transplantation, the patient remains alive and well with stable allograft function and no complication.

**Case 2**

**Figure 2:**
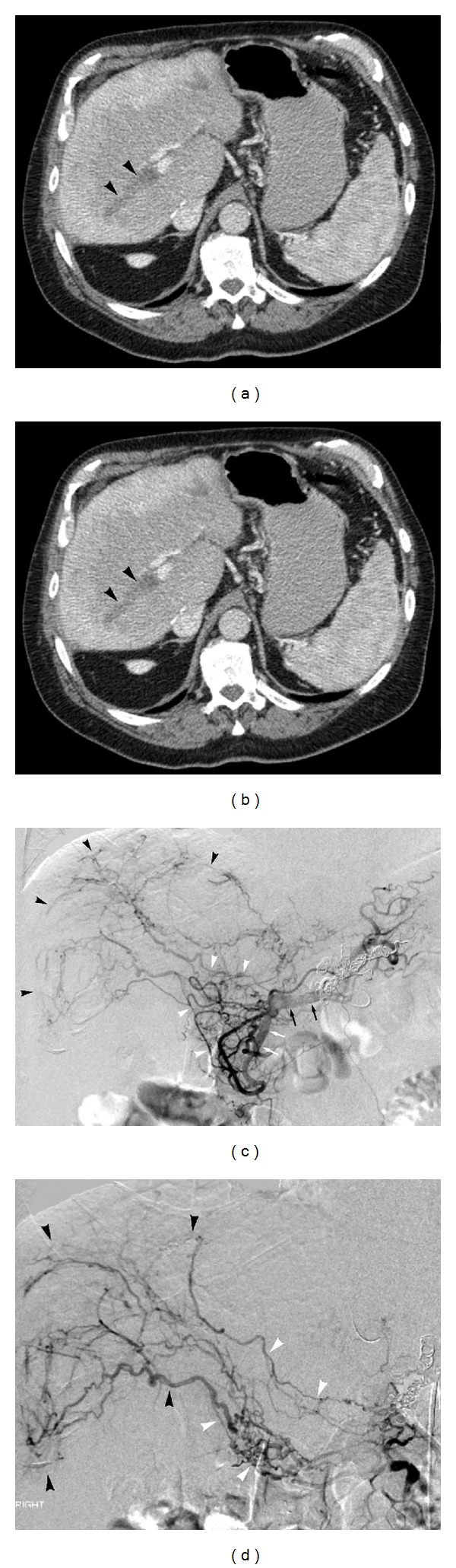
Axial contrast enhanced CT scan image (a) reveals right-sided intrahepatic portal vein thrombosis (arrowheads). Thrombus (arrowhead) extends into main portal vein on more caudal image (b). Subtracted common hepatic arteriogram (a) demonstrates chronic proper hepatic artery occlusion with transplant liver perfusion (black arrowheads) via mature pancreaticoduodenal collateral vessels (white arrowheads). Black and white arrows delineate common hepatic artery and gastroduodenal artery, respectively. Selective gastroduodenal arteriogram (b) better demonstrates mature pancreaticoduodenal collateral vessels (white arrowheads), which result in transplant liver perfusion (black arrowheads).

A 66-year-old male underwent OLT for alcoholic cirrhosis. Operation was done in a piggy bag fashion without venovenous bypass. Bile duct continuity was restored by duct-to-duct anastomosis. Patient had an uneventful postoperative course and two years later developed cyclosporine induced kidney failure for which he received a living donor kidney transplant. The patient also had recurrent episodes of lower extremity DVT and was on warfarin therapy. Six years later he was admitted to undergo colonoscopy for bleeding per rectum and warfarin therapy was withheld in preparation for colonoscopy. Simultaneously patient developed some derangement of liver function and underwent duplex ultrasonography and MRI of liver, which revealed a homogenous hepatic lesion at the dome of liver, intrahepatic portal vein thrombosis, and patency of hepatic artery but a slurring of systolic upstroke. Tumor markers were normal (AFP = 5.4 mcg/L, CEA = 0.6 mcg/L). Thrombophilic workup was unrevealing. Biopsy of the liver lesion revealed normal liver architecture and no rejection. In view of extensive portal vein thrombosis as shown in Figures 2(a) and 2(b), a catheter directed thrombolysis with TPA was done with only partial improvement in flow which was short lasting and was complicated by progressive thrombosis of PV. The thrombolysis was followed by mild transaminitis and a pseudoaneurysm formation in the right lobe of liver. In order to delineate this anatomically a celiac angiogram was done which revealed complete occusion of common hepatic artery of the aloograft. Innumerable small collaterals arising from gastroepiploic artery and gastroduodenal artery were seen supplying the liver reconstituting intrahepatic arterial supply as shown in Figures 2(c) and 2(d). Serial multiple follow-up DUS and MRI confirmed the blockage of dual blood supply to liver. During this entire course the liver function stayed completely normal. Three years after dual thrombosis, the patient is alive and well with stable allograft function and no complication.

## 3. Discussion

Complete devascularization of the native liver is difficult to achieve due to the dual blood supply and number of collaterals which develop in case of vascular interruption. Total hepatectomy at the time of transplant disrupts these collaterals; however, allograft may survive only on the portal flow while arterial collaterals develop over few weeks [8]. When the complete dearterialization of native liver is achieved experimentally, extensive necrosis is not averted by intact portal circulation [9] and an anecdotal series has shown death rate in excess of 50% with accidental hepatic artery ligation [10]. In contrast, a recent report in which the hepatic artery was ligated for HA rupture following liver transplantation found that all 6 patients with HA ligation were alive, though some patients required adjunctive procedures [11]. Another anecdotal report by Tygstrup et al. showed that, with occlusion of the proper hepatic artery, extraction of oxygen from the portal venous blood increased, thereby compensating for the loss of the arterial supply. They concluded that, under ordinary circumstances, the hepatic artery could be ligated with impunity [12].

The foregoing data have made it clear that an effective compensatory mechanism of increased oxygen extraction from the portal venous blood is ordinarily sufficient to maintain hepatic viability. It suggested that many of the fatalities which have followed interruption of the HA are probably due to factors which either have further increased hepatic oxygen needs or have reduced the volume of portal flow like fever, atelectasis, cardiac failure, and shock.

Similarly in case of interruption of portal blood flow due to PVT, which accounts for two-thirds of total liver blood flow, the liver allograft has the tendency to survive in most of the cases due to arterial buffer response and rapid development of collaterals, which become apparent in several days, reconstituting the intrahepatic blood flow [13].

We believe that the predisposing factors for vascular thrombosis in our first patient were generalized hypercoagulable state due to previous malignancy, the presence of aortohepatic conduit, and surgical reconstruction of portal vein and portal venous thrombectomy as shown by others [3, 14]. Of note, because of the increased risk of posttransplant vascular complications due to preoperative radiotherapy, aortohepatic conduit using cadaveric iliac artery graft was recommended to avoid late arterial complications. Nonetheless, any modification of portal vein reconstruction has not been recommended because large veins are less damaged by irradiation [15]. It has been reported that infrarenal aortohepatic conduit is an effective option for reconstruction of the hepatic artery of the transplant liver and the long-term graft survival is comparable to that of control group, despite the higher incidence of HAT [16]. The second patient had percutaneous biopsy of the liver and subsequently TAE of hepatic pseudoaneurysm as a sequel of biopsy. We believe that TAE of pseudoaneurysm resulted in traumatizing hepatic artery resulting in increased periarterial inflammation, friability, and predisposition to thrombosis as has been reported previously [3]. Furthermore, during these invasive procedures accidental cannulation of portal vein changed the hemodynamics of portal system and these treatments triggered thrombosis similar to a previous report [17]. It is difficult to know the pathophysiology and effects of concurrent HA and PVT on morbidity and mortality following OLT due to paucity of such cases. After extensive search we are able to retrieve cases as shown in Table 1.

In our two cases, the concurrent HA and PVT were detected during the follow-up and the liver function was pristine. The blood flow to the liver was reconstituted by various portal and arterial collaterals. Neovascularization of the ischemic liver is an obscure phenomenon and various mechanisms have been proposed. Some ascribe a role to angiogenic properties of omentum and mesentery [20]. Others proposed opening and dilatation of vasa vasorum along the hepatic artery and capillaries and venules around the portal vein. Since during the implantation of liver all of these connections are severed, it has been proposed that this neovascularization may occur de novo and the precise mechanisms are not fully understood. Experiments of hepatic ischemia in murine model lead to upregulation of VEGF, a key factor which triggers neovascularization. Moreover exogenous recombinant VEGF can induce angiogenesis in a host [21]. Furthermore, correlation between prolonged hepatic hypoxia and angiogenesis was found by Panaro et al. implying an association between the HAT and neovascularization [22]. In the transplant setting, ischemic liver could trigger an enzymatic cascade for angiogenesis leading to neovascularization from surrounding arterial and venous territories [21].

In the present two cases we opine that neovascularization might have been triggered by the ischemic liver late in the posttransplant period as the liver got ischemic or the neovascularization might have occurred time ahead of the dual thrombosis permitting the survival of allografts. Robustness of the collateral circulation was elucidated by angiography in both of these cases. Neovascularized hepatic allografts that had suffered late HAT and PVT may survive, though there remains an increased risk of development of ischemic cholangiopathy [22].

## 4. Conclusion

Concurrent HAT and PVT early in the aftermath of OLT are devastating complications leading to loss of allograft and necessitating retransplantation. However if the event occurs late, simple observation may suffice provided that the robustness of collateral circulation and reconstitution of intrahepatic blood flow are elucidated angiographically. Nonetheless, these allografts should have more rigorous surveillance due to high risk of ischemic cholangiopathy.

## Figures and Tables

**Table 1:** Depiction of cases with concurrent HAT and PVT in the literature.

Author (year)	Number of patients/type	Indications for transplant	Type of transplant	HAT and PVT in relation to transplant	Treatment	Outcome
Langnas et al. (1991) [1]	3/2 children, 1 adult	Biliary atresia 2, cholangiocarcinoma 1	CLTx	Within a week	Adult Re-Tx, 1st child Re-Tx, 2nd child observation	2 deaths, 1 child in vegetative state at 4 years

Kaneko et al. (2004) [7]	4/2 children, 2 adults	Biliary atresia 1, Wilson's disease 1, PBC 1, primary hyperoxaluria 1	LDLTx	Within a week	3 thrombectomy, 1 RE-Tx	4 deaths

Haque et al. (2009) [18]	1 adult	Wilson's disease 1	CLTx	12 years	Conservative	Alive at one year after the event

Ayala et al. (2011) [19]	1 adult	NA	CLTx	NA	NA	NA

Present series (2013)	2 adults	Cholangiocarcinoma 1, alcoholic cirrhosis 1	CLTx	6 months 6 years	Conservative	Alive with normal liver function at 3 years

CLTx: cadaveric liver transplant, LDLTx: living donor liver transplant, Re-Tx: retransplantation, NA: not available.

## Abbreviations

HAT: Hepatic artery thrombosis
PVT: Portal vein thrombosis
OLT: Orthotopic liver transplantation
TAE: Transarterial embolization
HA: Hepatic artery
PV: Portal vein
PBP: Peribiliary plexus
ITBL: Ischemic type biliary lesions
DUS: Doppler ultrasound
DVT: Deep venous thrombosis.
